# Is Baseline Cardiac Autonomic Modulation Related to Performance and Physiological Responses Following a Supramaximal Judo Test?

**DOI:** 10.1371/journal.pone.0078584

**Published:** 2013-10-18

**Authors:** Cristina Blasco-Lafarga, Ignacio Martínez-Navarro, Manuel Mateo-March

**Affiliations:** 1 Physical Education and Sports Department, University of Valencia, Valencia, Spain; 2 University Miguel Hernandez, Elche, Alicante, Spain; Universidad Europea de Madrid, Spain

## Abstract

Little research exists concerning Heart Rate (HR) Variability (HRV) following supramaximal efforts focused on upper-body explosive strength-endurance. Since they may be very demanding, it seems of interest to analyse the relationship among performance, lactate and HR dynamics (i.e. HR, HRV and complexity) following them; as well as to know how baseline cardiac autonomic modulation mediates these relationships. The present study aimed to analyse associations between baseline and post-exercise HR dynamics following a supramaximal Judo test, and their relationship with lactate, in a sample of 22 highly-trained male judoists (20.70±4.56 years). A large association between the increase in HR from resting to exercise condition and performance suggests that individuals exerted a greater sympathetic response to achieve a better performance (Rating of Perceived Exertion: 20; post-exercise peak lactate: 11.57±2.24 mmol/L; 95.76±4.13 % of age-predicted HR_max_). Athletes with higher vagal modulation and lower sympathetic modulation at rest achieved both a significant larger ∆HR and a faster post-exercise lactate removal. A enhanced resting parasympathetic modulation might be therefore related to a further usage of autonomic resources and a better immediate metabolic recovery during supramaximal exertions. Furthermore, analyses of variance displayed a persistent increase in α_1_ and a decrease in lnRMSSD along the 15 min of recovery, which are indicative of a diminished vagal modulation together with a sympathovagal balance leaning to sympathetic domination. Eventually, time-domain indices (lnRMSSD) showed no lactate correlations, while nonlinear indices (α_1_ and lnSaEn) appeared to be moderate to strongly correlated with it, thus pointing to shared mechanisms between neuroautonomic and metabolic regulation.

## Introduction

During the last decade, Heart Rate (HR) Variability (HRV) has been widely used as a non-invasive marker of the Autonomic Nervous System (ANS) regulation of HR dynamics following exercises of different duration and intensities. Recently, new studies have been conducted to analyse cardiac autonomic regulation following exercise intensities described as supramaximal, since interval protocols (i.e. repeated bouts of brief supramaximal exercise,~15-30s, interspersed with recovery periods of similar duration) appear to be extremely effective in already highly-trained endurance athletes [1,2]; also in combat sports training [3]. 

Buchheit et al. [4] formerly showed a significant slower parasympathetic reactivation after a Repeated Sprint exercise compared with a continuous one of equivalent net caloric expenditure, concluding that anaerobic metabolism participation, rather than energy expenditure, modulates the level of post-exercise parasympathetic reactivation. A similar depressed post-exercise cardiac autonomic response has been reported by other authors following the anaerobic Wingate Test [5-8], Repeated Shuttle Sprints [9], or varying protocols of interval running exercises [10], suggesting that the depression of parasympathetic HRV indices (i.e., high frequency power, HF; and root-mean-square difference of successive normal R-R intervals, rMSSD) appears to be a common response to exercises with a large anaerobic component. 

Since all of the aforementioned studies have employed efforts mainly focused on lower limb, little research exists concerning cardiac autonomic regulation following supramaximal efforts, thus anaerobic, requiring high both neuromuscular and cardiovascular demands on the upper body; which it is a characteristic profile of some fighting sports like Judo [11-13]. Although these sports require the concomitant metabolic involvement of both anaerobic and aerobic pathways, the anaerobic capacity, focused in the upper body, points to be the prevalent and determining capacity due to the explosive throwing actions, as well as the gripping and grappling continuous actions in the contest [12,13]. 

On the other hand, it has been described higher lactate peaks in top athletes as an adaptive response to high intensity training [14,15]; but also a better lactate removal ability; this last due to an enhanced maximal muscle oxidative capacity and a greater content of monocarboxylate transporters [16,17]. This higher lactate clearance capacity, related in turn to a delayed appearance of muscle fatigue [18,19], might become a confounding factor in the assessment of the anaerobic capacity in the above cited multi-acceleration sport tasks [20,21]. However, despite this clearance capacity, the resulting stress metabolite accumulation importantly influences post-exercise sympathetic inhibition timeline through chemoreflex control of HR [22,23], thus interrelating metabolic and cardiac autonomic recovery processes.

To our knowledge, no previous study had undertaken a comprehensive analysis integrating: baseline cardiac autonomic modulation, HR indices (i.e., maximal HR increase during exercise and HR decay during recovery), performance, post-exercise blood lactate (i.e., peak accumulation and clearance) and nonlinear HR dynamics during the immediate recovery from an intervallic supramaximal exertion, mainly focused in upper limb strength. Given this scenario, the Blasco Specific Judo Test [BSJT;12], a Judo test which includes upper limb strength exercises, offers a validated [24] and suitable proposal to assess the relationship between cardiac autonomic control and lactate dynamics following intervallic supramaximal exercises primarily aimed at upper body, which have not yet been addressed in HRV studies. 

Therefore, the present study aims: (1) to examine possible associations between: short-term baseline measures of HRV, performance, and both HR and blood lactate responses during and following a supramaximal judo test; (2) and to compare HRV and lactate dynamics during its immediate recovery. Following Nunan [25], we hypothesize that post-exercise HR dynamics might be related to baseline cardiac autonomic regulation. We also hypothesize an important mediating role for anaerobic metabolism handling (i.e., lactate accumulation and clearance) in post-exercise nonlinear HR dynamics.

## Methods and Design

### Participants and experimental protocol

Twenty two male judokas from under-60 to under-81 kg categories (20.70 ± 4.56 years, 1.73 ± 0.06 m, 68.25 ± 7.17 kg, 22.84 ± 1.64 kg*m^-1^), took part in the study. They all had 3 to 7 years of competitive experience at senior national and international level and trained for 13 ± 6 hours a week. The study conformed to the Declaration of Helsinki and participants gave voluntary written consent to participate in the investigation, approved by the ethics committee of the University of Valencia. In the event of underage athletes, coaches signed a consent statement indicating that they have been advised of the potential dangers of participation and that they had understood them. The underage written consent also included the judokas’ signature to show that they also acknowledged of these terms [26]. 

Special care was taken to standardize tapering prior to the test. Vigorous exercise was not allowed for 48 h before the testing day, and no training was permitted for 24 h before the testing day. Participants were summoned at 8:00 am on a fasting condition. On arrival to the gymnasium, they rested quietly for 10 min in the seated position to obtain baseline heart-rate derived data. They were allowed to breathe spontaneously but reminded to avoid irregular respiration. Breakfast was instructed to be appropriate for the subsequent exertion, and 2 h later a standardized warm-up was conducted to prepare participants for BSJT. Immediately after the test (<5 s), the judokas were assisted to sat passively on a chair placed adjacent to the tatami and remained still for the following 15 min. Sitting position and spontaneous breathing were chosen in order to match baseline and post-exercise conditions, as lying down position and controlled breathing would have been impractical during recovery. Moreover, normal respiratory rate does not result in significantly different heart rate-derived indices compared with controlled breathing [27].

### Performance assessment: The BSJT

The assessment of sport performance and the associated physiological profile remains under debate in most complex anaerobic sports [28], since activity patterns are very changing and considerably influenced by the opponent features [21]. Besides, there is the issue of the specific nature of the sport and the ecological validity of some classical laboratory variables such as the maximum oxygen consumption attained in a treadmill or a cycloergometer maximal graded test [29]. In this context, since Judo contest cannot always be considered as a reference of maximum exertion, BSJT is aimed at reproducing both neuromuscular and energy demands of judo competition within a time structure similar to that of a contest, in order to assess the judoist’s maximum performance and the use of specific skills under fatigue conditions. Time structure and the drills employed in the test (pull-ups, gripping up and down a rope, and four Judo specific tasks) have been previously described in detail, and the test have already shown to be valid (with significant performance differences between elite and national level judoist; n=53, 29 male and 24 female; p<0.05 for both, male and female athletes) [12]; and reliable (Test-Retest Intraclass Correlation 0.875; p = 0.003) [24]. 

Briefly, BSJT comprises six 15-s exercise periods, separated by either 10-s or 15-s recovery periods, repeated for three consecutive bouts (i.e., eighteen periods of 15-s maximum intensity); then a larger 30-s recovery period and a final “time-to-exhaustion” isometric pull-up (_TE_PU, see Table 1 for a glossary of abbreviations). This final exercise assesses judoist’s upper limb strength reserve (i.e., residual isometric strength of pulling muscles) following a series of strenuous explosive-strength efforts; a strength reserve that may become crucial in the Golden Score extra-time of Judo contests. Accordingly, total duration of the test varies from 8 to 9 minutes, depending on each judoist’s capacity to maintain the _TE_PU. Because the BSJT assesses some specific judo drills, three judokas of similar body weights are needed to perform the test: 1 participant is evaluated and 2 other individuals receive throws. Finally, BSJT offers seven different performance assessment sub-scores: whole number of repetitions performed in each of the six drills and time achieved in the final _TE_PU, which after being converted into t-scores, are added up to generate a global performance score (BSJT_score_). 

**Table 1:** Glossary of abbreviated terminology.

**Performance indices and recovery periods (see also Figure *1*)**	
_TE_PU	Time-to-exhaustion Isometric Pull-Up
BSJT_score_	Sum of T-scores for the seven skills involved in the *Blasco Specific Judo Test*
RPE	Rating of Perceived Exertion
Post1	Recovery period comprised between 180 and 360 s after *Blasco Specific Judo Test*
Post2	Recovery period comprised between 360 and 540 s after *Blasco Specific Judo Test*
Post3	Recovery period comprised between 540 and 720 s after *Blasco Specific Judo Test*
Post3	Recovery period comprised between 720 and 900 s after *Blasco Specific Judo Test*
**Cardiovascular and metabolic terms and indices**	
HR_baseline_	Baseline heart rate
HR_final_	Heart rate at the end of *Blasco Specific Judo Test*
HR_max_	Maximum heart rate achieved during *Blasco Specific Judo Test*
ΔHR	Difference between maximum heart rate achieved during the test and baseline heart rate
HRR_1_	Difference between final heart rate and heart rate following 1 min of recovery
HRR_2_	Difference between final heart rate and heart rate following 2 min of recovery
HRV	Heart Rate Variability
lnSDNN	Log-transformed Standard Deviation of all normal RR intervals
lnRMSSD	Log-transformed the Root-Mean-Square difference of successive normal RR intervals
lnHF	Log-transformed High Frequency power
lnLF	Log-transformed Low Frequency power
lnLFHF	Log-transformed Low Frequency to High Frequency ratio
α_1_	Short-term fractal scaling exponent
lnSaEn	Log-transformed Sample Entropy
[La]_n_	Lactate concentration, where n= min of recovery (i.e. 1,3,5,10 & 15 min)
[La]_peak_	Lactate Peak concentration
[La]_cle_	Difference between [La]_15_ and [La]_peak_

### HR, HRV, RPE and blood lactate concentrations measurement

HR and Rating of Perceived Exertion (RPE, scale 6-20) [30] were obtained immediately after the BSJT as measures of the relative intensity achieved by each subject. Final HR was retained for analysis as an absolute value (HR_final_) and as a percentage of the age-predicted maximum HR (%HR_final_). Recordings were performed using a Polar RS800 HR monitor set to RR interval mode (Polar Electro, Kempele, Finland) together with an electrode transmitter belt (Polar Wearlink Wind, Polar Electro, Kempele, Finland), after application of conductive gel as recommended by the manufacturer. Moreover, prior to the test, to prevent the electrode from coming unstuck, transmitter belt was firmly held by adjusting a sticky bandage around the thorax. Data were transferred to Polar Pro Trainer 5 software (Polar Electro, Kempele, Finland) and afterwards analysed by means of Kubios HRV Analysis Software 2.1 (The Biomedical Signal and Medical Imaging Analysis Group, Department of Applied Physics, University of Kuopio, Finland). Baseline data were obtained from the last 5-min segment of the resting recording, whereas recovery data were extracted on four 180-s windows (Post1, Post2, Post3 and Post4). The initial 180 s were not evaluated to minimize the influence of pronounced downward trends [31]. 5-min segments during resting recordings were employed to equate as much as possible the number of data points analyzed in resting and recovery conditions.

**Figure 1:**
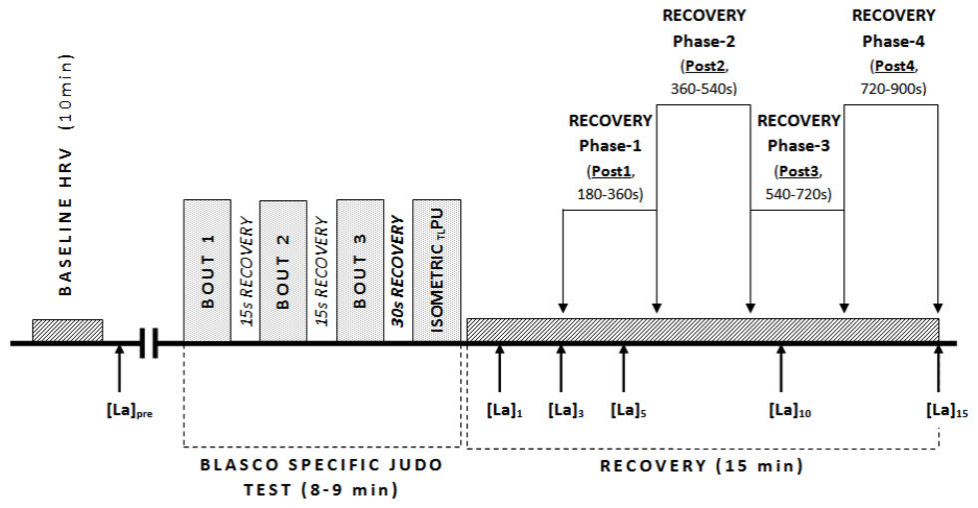
Schematic view of the experimental protocol.

Immediately before the warm-up, and 1, 3, 5, 10 and 15 minutes following BSJT, an earlobe capillary blood sample was collected for lactate concentration analysis ([La]_pre_, [La]_1_, [La]_3_, [La]_5_, [La]_10_, [La]_15_) using a Lactate Pro LT-1710 analyser (Arkray Inc, Kyoto, Japan) [32]. The maximum value reached during the recovery period was defined as the peak concentration ([La]_peak_), whereas [La]_cle_ was calculated by subtracting lactate concentration at the 15^th^ minute of recovery from [La]_peak_, see Figure **1**. 

### HR and HRV data analysis

The absolute change between baseline HR (HR_baseline_) and maximum HR achieved during the test was defined as ∆HR; whereas HRR_1_ and HRR_2_ were calculated by subtracting HR recorded following 1 and 2 min of recovery from HR_final_. In these latter cases, HR was obtained as a mean value of a 5-s window. 

The whole analysis process was carried out by the same researcher to ensure consistency. Artifacts were identified and corrected, and resting recordings were also detrended (Smooth priors, ƛ = 500) according to manufacturer’s recommendations. Regarding linear analysis during resting condition, the standard deviation of normal RR intervals (SDNN) and rMSSD were calculated for the time domain. Low frequency power (LF, 0.04-0.15 Hz) and High Frequency (HF 0.15-0.4 Hz) were calculated as integrals of the respective power spectral density curve, using a Fast Fourier Transform algorithm; and LF/HF ratio was also retained for statistical analysis. 

Examination of nonlinear HR dynamics was completed using measures of fractal scaling properties and complexity. Detrended Fluctuation Analysis (DFA) technique was applied to quantify self-similarity correlations [33]. Based on previous research and because of our relatively short recording time, only the short-term (4 to 11 beats) scaling exponent (α_1_) was retained for statistical analysis. Eventually, Sample Entropy (SaEn) was calculated to provide a general indication of predictability of the time-series [34]. To characterize the stringency of match recognition, the length (m) of the subseries and the tolerance (r) of the matches were fixed at m = 2 and r = 20% of the SD of the datasets. 

Later on, during recovery condition, only rMSSD was considered within linear indices, in order to measure the vagal reactivation [35], while frequency-domain indices were discarded given that its reliability is worsened following supramaximal efforts [36]. Nonlinear indices completed the analysis: α_1_ is considered to estimate sympathovagal balance [37], and SaEn to provide an indication of the complexity of the time-series under these circumstances [34].

### Statistical Analyses

Statistical analyses were carried out using the Statistical Package for the Social Sciences software (SPSS version 15.0, SPSS Inc., Chicago, USA). After testing for normal distribution (Kolmogorov-Smirnov test, with Lilliefor’s correction), SDNN, RMSDD, LF, HF, LF/HF and SaEn were logarithmically transformed to allow parametric comparisons. Those variables will henceforth be referred as lnSDNN, lnRMSSD, lnHF, lnLF, lnLFHF and lnSaEn respectively. 

Possible relationships between HR_baseline_, HRV indices and BSJT_score_, heart rate measures (∆HR, HRR_1_, HRR_2_), and post-exercise lactate concentrations ([La]_peak,_ [La]_cle_), were initially examined using a Pearson correlation. Besides, considering the previously described strong dependence of resting HRV indices on age [38] and intrinsic HR [39], and also on total spectral power in the case of linear measures [31], this analysis was re-conducted using a partial correlation controlling for either these three or two variables. A second Pearson correlation analysis was later performed to assess possible associations between BSJT_score_, heart rate measures (∆HR, HRR_1_, HRR_2_) and post-exercise lactate concentrations ([La]_peak,_ [La]_cle_). Eventually, a third partial correlation analysis, now controlling for age, was performed to examine possible relationships between lnRMSSD, α_1_, lnSaEn and lactate concentrations during recovery, as sampling conditions matched similar time-windows (i.e. [La]_3_, [La]_5_,[La]_10_ and [La]_15_ vs. lnRMSSD, lnSaEn and α_1_ at Post1, Post2, Post3, and Post4). According to Cohen [40], correlations >0.5 are considered large, 0.3-0.5 are considered moderate and 0.1-0.3 are considered small.

Finally, a repeated measures multivariate analysis of variance, controlling for age and followed by Bonferroni post-hoc test, was used to assess the effect of the effort on lnRMSSD, lnSaEn and α_1_ (i.e. Baseline, Post1, Post2, Post3, and Post4) and lactate (i.e. Baseline, 3, 5, 10 and 15 minutes after the test). Whenever Mauchly’s Sphericity test was violated, necessary technical corrections were performed using the Greenhouse-Geisser test. Partial estimated effect size (partial η2) was reported as a measure of the magnitude of the effect. The significance level was set at p<0.05. However, trends towards signification (p<0.1) were also named [41]. Data are presented as means and standard deviations (±SD). 

## Results

Artifact beats accounted for less than 1% in the 22 participants presented in the study. Descriptive data of baseline HRV, performance, RPE at the end of the test, HR indices and lactate concentration measures are presented in Table 2. 

**Table 2:** Descriptive data (mean ± SD) of resting HRV, performance, RPE, HR and lactate measures following BSJT.

**Cardiovascular and anthropometric baseline variables**		**Performance RPE, HR and lactate measures at BSJT**	
**Age** (years)	20.70 ± 4.56	**BSJT**_score_	303.26 ± 42.94
**IMC** (kg/m^2^)	22.84 ± 1.65	_TE_ **PU**(s)	28 ± 8.23
**HR** (beats)	60.92 ± 11.78	**RPE**_final_	20 ± 0
ln**SDNN** (ms)	4.16 ± 0.52	**HR**_max_ (bpm)	193.67 ± 9.57
ln**RMSSD** (ms)	4.16 ± 0.66	%HR_max_	95.76 ± 4.13
ln**LF** (ms^2^)	7.38 ± 1.28	∆**HR** (beats)	133.31 ± 10.96
ln**HF** (ms^2^)	7.27 ± 1.22	**HRR**_1_ (beats)	40.80 ± 12.13
ln**LF/HF** (ratio)	0.11 ± 1.14	**HRR**_2_ (beats)	56.66 ± 11.63
α_1_	0.97 ± 0.39	[La]_peak_ (nmol/L)	11.57 ± 2.24
Ln**SaEn**	0.42 ± 0.32	[La]_cle_ (mmol/L)	1.91 ± 1.79

Abbreviations are explained in Table 1.

### Correlation analyses

**Figure 2:**
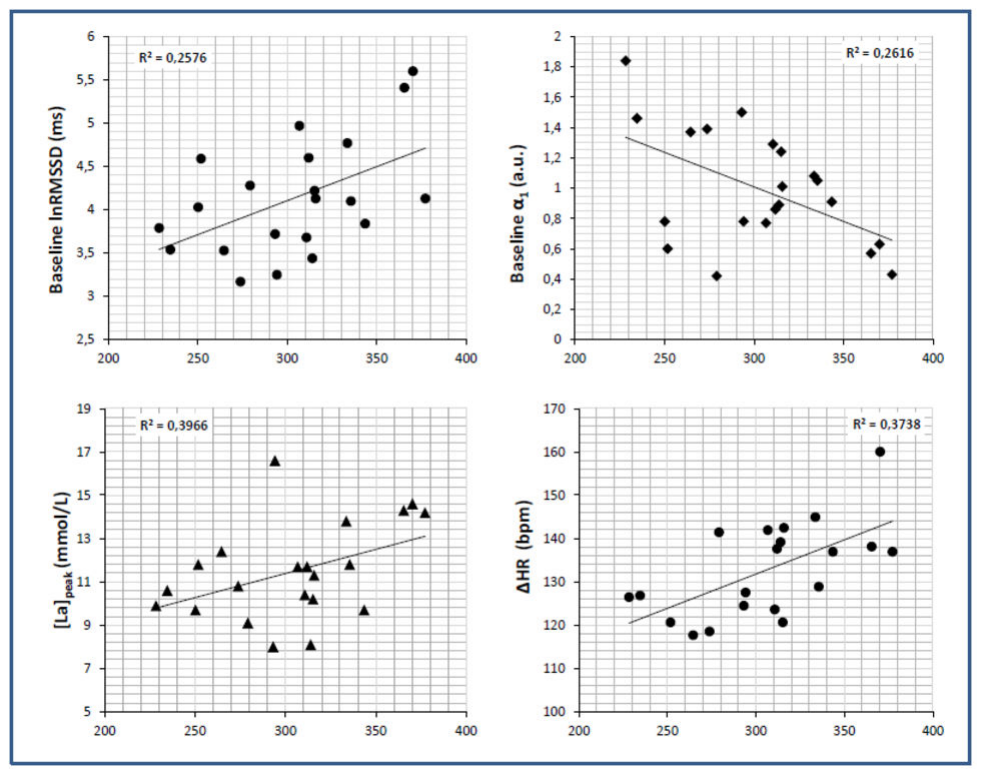
Relationship between BSJT_score_ (*x-axis*) and different variables (*y-axis*): [La]_peak_, ΔHR, lnRMSSD and α_1_. Linear regression (R^2^) is included.

BSJT_score_ was largely and positively correlated to ∆HR (r = 0.61). It was borderline to significance with [LA]_peak_ (p = 0.053; r = 0.43), and showed a moderate inverse correlation with HRR_2_ (r = -0.45) (Table 3). In addition, it was moderate to largely and positively correlated to baseline lnRMSSD (r = 0.52) and lnHF (r = 0.47), and largely and negatively associated with α_1_ (r = -0.58). However, after controlling for confounding variables, the correlation with lnHF disappeared, and those with lnRMSSD and α_1_ slightly decreased and became trends (lnRMSSD: p = 0.057; r = 0.47; α_1_: p = 0.055; r = -0.45), but emerged a moderate inverse association with HR_baseline_ (r = -0.46) (Table 4). Regarding [LA]_peak_, it was largely and inversely correlated with both HRR_1_ and HRR_2_ (r = -0.60 and r = -0.55, respectively) (Table 3). Besides, it showed an association with HR_baseline_ (r = -0.47) and a trend to correlation with baseline α_1_ (p = 0.059; r = 0.43), but both relationships disappeared in the partial correlation (Table 4). Concerning [LA]_cle_, it was unrelated to BSJT_score_ and HRR measures (Table 3); however, the partial correlation with baseline HRV measures showed a moderate association with lnHF (r = 0.50), lnLFHF (r = -0.49) and α_1_ (r = -0.48) (Table 4). On the other hand, ∆HR appeared moderate to largely correlated with HR_baseline_ (r = -0.62), lnSDNN (r = 0.50), lnRMSSD (r = 0.67), lnHF (r = 0.65), lnLFHF (r = -0.51) and α_1_ (r = -0.56) in the Pearson correlation; but only the association with HR_baseline_ (r = -0.58) and lnHF (r = 0.51) remained significant in the partial correlation, although the correlation with lnLFHF (p = 0.53; r = -0.48) was also nearly significant (Table 4). Besides, ∆HR moderately and inversely correlated with HRR_2_ (r = -0.47) (Table 3). At the same time, both HRR_1_ and HRR_2_ were largely and positively associated with HR_baseline_ (value range = 0.61 to 0.65), remaining significant the relationship with HRR_1_ in the partial correlation (r = 0.51) and trend to significant with HRR_2_ (p = 0.084; r = 0.41). Both recovery indices appeared also moderately and inversely correlated with baseline lnRMSSD in the Pearson correlation (value range = -0.46 to -0.48), but the relationships disappeared after controlling for confounding variables. Notwithstanding, in this partial correlation HRR_2_ appeared significantly and inversely correlated with lnSDNN (r = -0.50) and a trend towards significance emerged with lnSaEn (p = 0.059; r = 0.45). Eventually, HRR_1_ and HRR_2_ were largely and positively correlated to each other (r = 0.90). The results of this first and second subset of correlation analyses are presented in Table **3** and Table **4**. Figure **2** illustrates using scatter plots some main significant correlations regarding BSJT_score._

**Table 3:** Correlations between performance and physiological responses to exercise.

	**BSJT_score_**	**∆HR**	**HRR_1_**	**HRR_2_**	**[La]_peak_**	**[La]_cle_**
**BSJT_score_**	-	0.611**	-0.399**^*#*^**	-0.446*	0.428**^#^**	0.211
**∆HR**	0.611**	-	-0.384**^*#*^**	-0.472*	0.308	0.045
**HRR_1_**	-0.399**^*#*^**	-0.384**^*#*^**	-	0.890**	-0.595**	-0.077
**HRR_2_**	-0.446*	-0.472*	0.890**	-	-0.548*	-0.048
**[La]_peak_**	0.428**^#^**	0.308	-0.595**	-0.548*	-	0.240
**[La]_cle_**	0.211	0.045	-0.077	-0.048	0.240	-

Abbreviations explained in Table 1. ^#^ *p*<0.1; *p<0.05; **p<0.01.

**Table 4:** Pearson (*R*) and Partial correlations (*_P_R*) either controlling for Age (HR_baseline_), Age and RRi (α_1,_ lnSaEn) or lnTP, Age and RRi (lnSDNN, lnRMSSD, lnLF, lnHF and lnLF/HF) between baseline HRV measures (rows), and performance and physiological responses to exercise (columns).

	**BSJT**_score_		∆**HR**		**HRR**_1_		**HRR**_2_		[La]_peak_		[La]_cle_	
	**R**	***_P_R***	**R**	***_P_R***	**R**	***_P_R***	**R**	***_P_R***	**R**	***_P_R***	**R**	***_P_R***
**HR**_baseline_	-0.229	*-0.457* *	-0.617**	*-0.581* *	0.646**	*0.512* *	0.607**	*0.407* **^#^**	-0.472*	*-0.163*	-0.093	*-0.145*
ln**SDNN**	0.347	*0.128*	0.496*	*0.161*	-0.331	*-0.383*	-0.303	*-0.504* *	0.193	*-0.280*	0.223	*-0.100*
ln**RMSSD**	0.519*	*0.471* **^*#*^**	0.667**	*0.409*	-0.476*	*-0.240*	-0.457*	*-0.307*	0.378	*-0.007*	0.292	*0.436* **^*#*^**
ln**LF**	0.135	*-0.199*	0.175	*-0.401*	-0.158	*-0.215*	-0.116	*-0.146*	0.170	*-0.042*	0.109	*-0.446* **^*#*^**
ln**HF**	0.468*	*0.350*	0.649**	*0.514* *	-0.286	*0.133*	-0.275	*0.134*	0.393**^†^**	*0.175*	0.397**^†^**	*0.496* *
ln**LF/HF**	-0.358	*-0.286*	-0.511*	*-0.477* **^*#*^**	0.129	*-0.180*	0.190	*-0.146*	-0.234	*-0.115*	-0.311	*-0.490* *
**α_1_**	-0.576*	*-0.450* **^*#*^**	-0.564*	*-0.329*	0.342	*-0,061*	0.376	*-0.033*	-0.429**^*#*^**	*-0.180*	-0.347	*-0.480* *
ln**SaEn**	0.236	*0.110*	0.215	*0.023*	0.021	*0.357*	0.032	*0.452* **^*#*^**	0.393**^#^**	*0.241*	0.283	*0.346*

Abbreviations explained in Table 1. ^#^ *p*<0.1; *p<0.05; **p<0.01.

Table **5** displays the results of the third subset of correlation analysis, related to recovery time course. lnRMSSD showed no correlation at all. α_1_ showed an inverse, moderate to large correlation with lactate scores throughout the recovery period, with the exception of [La]_15,_ that was unrelated to α_1_ at any time window. On the other hand, lnSaEn was largely and positively associated with [La]_3_, [La]_5_ and [La]_10_ (r value range = 0.53 to 0.62) at Post1, but only the relationship with [La]_10_ remained significant at any other time windows (i.e., Post2 and Post4). Similarly to α_1_, [La]_15_ was not correlated with lnSaEn at any time period. 

**Table 5:** Partial correlations, controlling for age, between lactate concentration and lnRMSSD, lnSaEn and α_1_ during recovery. *The* *shadowed* *areas* *illustrate* *the* *time* *course* *of* *the* *relationships* .

	**[La]_3_**	**[La]_5_**	**[La]_10_**	**[La]_15_**
**lnRMSSD**_Post1	-0.147	-0.121	-0.168	-0.153
**lnRMSSD**_Post2	-0.132	-0.066	-0.135	-0.105
**lnRMSSD**_Post3	-0.129	-0.066	-0.107	-0.129
**lnRMSSD**_Post4	-0.128	-0.122	-0.165	-0.143
**α_1_**_Post1	-0.435*	-0.338	-0.442*	-0.112
**α_1_**_Post2	-0.500*	-0.363	-0.444*	-0.059
**α_1_**_Post3	-0.491*	-0.451*	-0.525*	-0.293
**α_1_**_Post4	-0.649**	-0.479*	-0.688**	-0.293
**lnSaEn**_Post1	0.528*	0.552**	0.624**	0.301
**lnSaEn**_Post2	0.284	0.291	0.486*	0.197
**lnSaEn**_Post3	0.213	0.324	0.373	-0.033
**lnSaEn**_Post4	0.285	0.220	0.505*	0.179

Abbreviations explained in Table 1. ^#^ *p*<0.1; *p<0.05; **p<0.01.

### Nonlinear HR dynamics and lactate time courses during recovery

**Figure 3:**
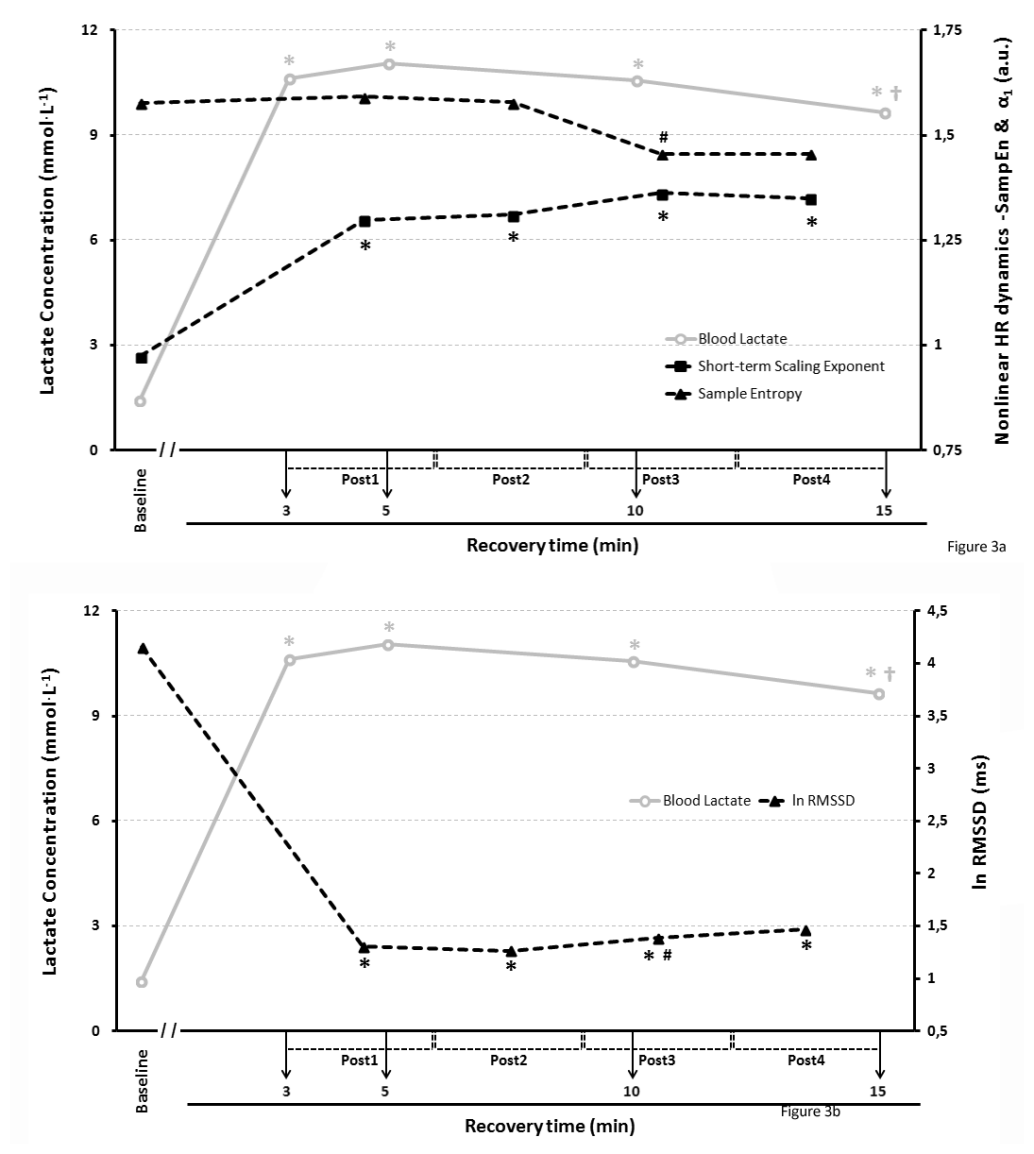
Heart Rate and lactate dynamics through the recovery period following BSJT. Nonlinear (Figure 3a) and Linear (Figure 3b) indices in black lines; lactate in a grey line. *Under figure*: * Significantly different from baseline condition (p<0.05 for α1; p<0.001 for lnRMSSD; p<0.01 for lactate); † Significantly different from [La]5. # Figure 3a: trend to significant difference from Post2 condition (p=0,056); # Figure 3b: Significantly different from Post2 condition (p<0.05).

Finally, univariate contrast analysis showed a significant effect for effort on lactate (F = 5.252; p = 0.006; η^2^ partial = 0.217; l-β = 0.856); lnRMSSD (F = 5.375; p = 0.024; η^2^ partial = 0.212; l-β = 0.656); and α_1_ [F = 4.579; p = 0.018; partial η^2^ = 0.194; l-β = 0.728)]; whilst lnSaEn displayed a non-significant and very small effect [F = 1.060; p = 0.354; partial η^2^ = 0.053; l-β = 0.216]. Further pairwise comparisons confirmed significant differences in lnrMSSD (p<0.001), α_1_ (p<0.05) and lactate (p<0.01) from baseline condition to any other sampling condition; as well as a significant increase in lnRMSSD from Post2 to Post3 and Post4 condition (p<0.05); and a significant difference between [La]_5_ and [La]_15_ (p<0.05), the highest and the lowest lactate scores during the recovery time period. In addition, despite the absence of a significant main effect for effort on lnSaEn, pairwise Bonferroni comparisons revealed a nearly significant decrease from Post2 to Post3 condition (p = 0.056), see Figure **3**. 

## General Discussion

Previous to the discussion of HRV and lactate results, it was important to ensure that the test was indeed a supramaximal upper limb effort. The values of RPE and HR at the end of the test confirmed this statement. [LA]_peak_ (11.57 ± 2.24 mmol/L) was close to the higher lactate productions described in previous studies based on judo combat simulation, i.e. 10.3 ± 2.6 and 9.2 ± 2.0 mmol/L for male and female Italian Olympic judoist [13], 10 mmol/L [42], or 12.3 ± 0.8 mmol/L [43]; and just a bit under lactate productions reached in real competition (13.4 ± 5.2 mmol/L in the first contest in an international judo tournament) [44]. However, although [LA]_peak_ during recovery from the BSJT test was also similar to those previously reported following usual running supramaximal intermittent protocols [4,9], it seemed slightly lower compared to those measured after repeated Wingate test [45,46]. Considering that the abovementioned studies were carried out with recreational athletes, which are known to reach lower [LA]_peak_ levels after supramaximal exercises [15,46], our lactate results seem even lower. A plausible explanation may lie in the fact that, even among specifically trained upper-limb athletes (i.e., judokas), it has been reported that the ability to perform anaerobic work with their upper body is only 81% of their lower limbs homologous capacity [47]. Additionally, it is plausible that during the 10 and 15-s recovery periods interspersed in the test, our athletes might have removed more lactate through aerobic pathways [17], given their higher fitness level (i.e., compared to recreational athletes).

### Performance, lactate, HR response and baseline cardiac autonomic modulation

In this anaerobic context, one of the major outcomes of the present study was that those athletes with higher vagal modulation (i.e., higher lnHF) and lower sympathetic modulation (i.e., lower HR) before the test, were capable of increasing their HR to a greater extent during the exercise protocol, which it is considered a measure of maximal sympathetic response [48]. This result is in contrast with those obtained by Nunan et al. [25], who found no association between baseline HRV and ∆HR. Nunan et al. [25] argued that resting HRV was only related to those HR responses that are vagally mediated (i.e., HR recovery indices). However, our findings are in accordance with Leeper et al. [48], who postulated that the absolute change from baseline HR to peak HR during a maximal exercise was connected to the level of basal vagal modulation. 

The association found between BSJT_score_ and ∆HR may be justified by the need to exert at the maximum individual capacity, thus the maximum sympathetic response, to optimize the performance in such a high-intensity intervallic test. Moreover, according to Halliwill et al. [49], the final isometric pull-up until exhaustion would have increased “the pressor response mediated by progressive, parallel increases in muscle sympathetic nerve activity and vascular resistance”. Similarly, resting lnRMSSD trend to correlation with BSJT_score_ may reinforce the idea that a higher baseline vagal modulation would allow further usage of autonomic resources for judokas’ responses to a supramaximal exercise, probably enabling a better performance during this kind of efforts. This postulate would be in accordance with the “autonomic resource hypothesis” proposed by Hynynen et al. [50]. These authors, by examining overtrained and non-overtrained athletes HRV response to a cognitive task, found that overtrained athletes were incapable of decreasing their HRV during a mental challenge (i.e., in relation to his individual resting values), unlike their non-overtrained counterparts. This absence of change (~response) was related to a worse performance in the task. Furthermore, since α_1_ matched lnRMSSD associations, it may be suggested that a sympathovagal balance leaning to vagal dominance (i.e., a lower α_1_) during resting conditions may contribute to a higher capacity to manage stress responses, and thus a better performance. 

A relevant issue may be the differences in time-domain (i.e. lnRMSSD) and frequency-domain indices (i.e. lnHF) when controlling for Age, RRi and lnTP. lnHF kept its significance in the partial correlation with ∆HR, which is also a cardiac index, while lost it regarding BSJT_score_., a measure of performance which is also mediated by factors unrelated to the cardiovascular functioning. Instead, lnRMSSD kept the trend to correlate with Performance, but lost its correlation regarding ∆HR, thus suggesting that the variables of control could be responsible for the pearson correlation with no controlls. Both, lnHF and lnRMSSD are vagal-mediated and intend to to measure the respiratory sinus arrhythmia [51] , but according to our results, the fast inter-beat changes in cardiac cycle measured by lnRMSSD might be better related to the overall factors that integrate performance. Although not exclusively, recent investigations point that power spectral analysis might provide a means to evaluate the ability to modulate autonomic outflows via baroreflexes [51,52] rather than the autonomic tone per se [51], what could explain some of these differences.

At the same time, and opposite to previous studies which found no relationship between baseline HRV and HRR indices [53-55] or even a positive association between resting parasympathetic activity and HR recovery post-exercise [25], the results of the present study have shown an inverse relationship between overall HRV (i.e., lnSDNN) at rest and HR recovery (i.e., HRR_2_) and a positive association among baseline and recovery HR indices following a supramaximal exertion. This discordance may be attributed to differences in the exercise protocols employed, since the individualized graded exercise protocols used in previous studies require a similar relative workload for each subject [48], whereas during supramaximal interval exercises, a better/worse handling of peripheral fatigue importantly mediates the timing of performance decay [18,19]. Namely, the better the capacity to maintain performance at high intensities (i.e., greater explosive strength-endurance), the greater the relative workload accumulated during a supramaximal interval exercise. Thereby, it seems that our inverse correlation between overall HRV at rest and HR recovery indices together with the positive association among baseline and recovery HR indices were highly mediated by the different relative workload exerted by each judoka, as BSJT_score_ was inversely correlated with HRR_2_. Large lactate productions during the test might explain the delay in recovery processes and thus the absence of correlations in regard to HRR_1_

Another novel finding of our study was the relationship found between baseline HRV and post-exercise lactate removal after controlling for the proper confounding variables. Those judokas who presented greater baseline vagal activity (i.e., higher lnHF) and a sympathovagal balance leaning to vagal dominance (i.e., lower α_1_ and lnLFHF) achieve a larger [LA]_cle_ during recovery. Therefore, it may be suggested that an enhanced vagal modulation and HR dynamics unpredictability during resting conditions could be related to improved lactate removal ability following high-intensity exercises, since Thomas et al. [18,19] demonstrated that lactate removal ability during recovery from supramaximal efforts keeps relationship with both the maximal oxidative capacity and the content of monocarboxylate transporters. Notwithstanding, further studies including biochemical measures are required to clarify whether this apparently improved metabolic capacity in those participants with an enhanced vagal modulation is related or not to better lactate removal mechanisms. 

### Post-exercise HR dynamics and lactate

Our results displayed a persistent significant increase in α_1_ and a decrease in lnRMSSD following BSJT (i.e., compared to baseline condition), which are indicative of diminished vagal modulation together with increased sympathetic modulation. These results are similar to previous studies assessing recovery from Wingate test [7], cycle ergometer incremental maximal exercise [56] and isometric handgrip [57]. The first two coincide with us in the anaerobic character of the effort, whereas we share with the latter the isometric handgrip ability. The increase in lnRMSSD from Post2 to Post3 condition shows a vagal modulation reactivation which, however, does not achieve returning cardiac parasympathetic activity to resting values. An unexpected absence of change in lnSaEn, from resting to post-exercise condition, suggest that HR complexity is affected to a different extent by a supramaximal effort, at least among highly-trained athletes, since previous studies involving non-athletes did show a decrease in lnSaEn during recovery [5,7]. Notwithstanding, these studies were conducted on very short lower-limb anaerobic efforts (i.e. wingate test), so further investigation is needed to clarify whether HR complexity following high-intensity exercise behaves differently among athletes and non-athletes, or it is related to differences in the protocol employed.. 

On the other hand, previous studies have also shown significant relationships between parasympathetic reactivation (both by means of HR recovery and HRV indices) and post-exercise blood lactate concentration [58,59]. Besides, recently Simoes et al. [60] have shown a concordance between HRV and lactate regarding anaerobic threshold determination. Our results corroborate these previous investigations [4,58,59] in relation to the fact that initial parasympathetic reactivation (i.e. HRR_1_ and HRR_2_) following exercises with a large anaerobic component is hindered by a delay in the inhibition of sympathetic activity, due to lactate effect on chemoreflex control of HR [22,23]. Actually, both HRR_1_ and HRR_2_ were strongly correlated with post-exercise [LA]_peak_. Furthermore, some significant, moderate to strong correlations found between lactate concentration and both SaEn and α_1_ during recovery (mainly considering the time course of the assessments; see the shadowed areas in Table **5**) suggest that sympathovagal balance and HR complexity following a supramaximal effort may be also influenced by lactate concentration. According to these correlations, those judokas with higher lactate concentrations in our study, exhibited a sympathovagal balance leaning to vagal dominance, jointly with more complex HR dynamics. This is in agreement with Tulppo et al. [61], who found that subjects who have a higher sympathetic activity following an exercise also exhibited an augmented vagal activity (i.e., post-exercise muscle sympathetic nervous activity was inversely associated with LF/HF ratio). Eventually, such greater interplay between vagal and sympathetic branches of the ANS would result in more complex HR dynamics.

Finally, unlike Obmiński et al. [44], who described a lactate clearance parabola following official Judo matches (2 to 5 min), our results displayed a lactate nearly steady-state, similarly to Bastos et al. [62]. As in this latter study, our athletes remained seated during the whole recovery period in order to match baseline conditions in regard to body position because this factor is known to significantly affect HR dynamics [63]. This seating position probably prevented our athletes from achieving a larger lactate removal [64], although the significant difference between [LA]_15_ and [LA]_5_ seems to indicate that athletes began to effectively remove lactate from blood from this moment on; unfortunately, we could not confirm this hypothesis as no further lactate samples were collected. 

### Limitations

The short follow-up and the absence of a control exercise with whom to compare HRV response obtained following BSJT makes necessary further studies that tackle with these issues. Another limitation of this study may be the employment of Polar HR monitors instead of ECG recordings to estimate RR intervals, as possible physiological artifacts, such as ectopic beats or arrhythmic events, are more difficult to detect. Notwithstanding, this instrument has been previously validated for the measurement of RR intervals and subsequent HR dynamics analysis [65,66]. Actually, Wallén et al. [67] recently reported that the agreement between RS800 HR monitor and electrocardiographic recordings range from good to excellent among men, thus reinforcing previous results from Weippert et al. [68]. Finally, spontaneous breathing may be another limitation, although this is a persistent limitation in those field-test studies following supramaximal efforts. Moreover, recent studies [69] have shown that there are no differences between spontaneous breathing and controlled breathing in the longitudinal follow-up of athletes, at least for RMSSD, which is an important index in our study.

## Conclusions

In summary, the major outcomes of this investigation were: (1) In highly-trained athletes, higher baseline vagal modulation and lower sympathetic activity prior to a supramaximal exertion is related to a greater response capacity during exercise, as evidenced by a larger ∆HR. Additionally, lower baseline sympathetic activity is also related to a better performance, and a clear positive tendency towards significance is present between resting vagal modulation and performance. These results endorse previous assumptions from Leeper et al. [48], and extend Hynynen et al. [50] related *Autonomic Resource Hypothesis* to supramaximal interval efforts (2). Following supramaximal exertions, initial HR decay is mediated by preceding individual relative workload limiting their use in inter-individual comparisons regarding parasympathetic reactivation capacity (3). An enhanced resting vagal modulation and a sympathovagal balance leaning to vagal dominance (i.e. lower α_1_) before performing a high-intensity exercise is related to an improved post-exercise lactate removal capacity. Future studies including biochemical measures should clarify whether this apparently improved metabolic capacity is related or not to better lactate removal mechanisms (4). Larger lactate concentrations after supramaximal upper limb explosive strength exercises are related, in our study, to a slower initial HR decay followed by a delayed sympathovagal balance leaning to vagal dominance and a more complex HR dynamics in highly trained judokas. Following Tulppo [61], long-lasting sympathetic hyperactivation due to lactate accumulation might have hindered initial HR recovery but subsequently enhanced cardiac sympathovagal outflow and complexity of HR dynamics. 

## References

[1] Laursen PB (2010) Training for intense exercise performance: high-intensity or high-volume training? Scand J Med Sci Sports 20 Suppl 2: 1-10. doi:10.1111/j.1600-0838.2009.00972.x. PubMed: 20840557. 20840557

[2] Laursen PB, Jenkins DG (2002) The scientific basis for high-intensity interval training: optimising training programmes and maximising performance in highly trained endurance athletes. Sports Med 32: 53-73. doi:10.2165/00007256-200232010-00003. PubMed: 11772161. 11772161

[3] Ravier G, Dugué B, Grappe F, Rouillon JD (2009) Impressive anaerobic adaptations in elite karate athletes due to few intensive intermittent sessions added to regular karate training. Scand J Med Sci Sports 19: 687-694. doi:10.1111/j.1600-0838.2008.00807.x. PubMed: 18694436. 18694436

[4] Buchheit M, Laursen PB, Ahmaidi S (2007) Parasympathetic reactivation after repeated sprint exercise. Am J Physiol Heart Circ Physiol 293: H133-H141. doi:10.1152/ajpheart.00062.2007. PubMed: 17337589. 17337589

[5] Goulopoulou S, Fernhall B, Kanaley JA (2009) Hemodynamic responses and linear and non-linear dynamics of cardiovascular autonomic regulation following supramaximal exercise. Eur J Appl Physiol 105: 525-531. doi:10.1007/s00421-008-0930-4. PubMed: 19034492. 19034492

[6] Mendonca GV, Heffernan KS, Rossow L, Guerra M, Pereira FD et al. (2010) Sex differences in linear and nonlinear heart rate variability during early recovery from supramaximal exercise. Appl Physiol Nutr Metab 35: 439-446. doi:10.1139/H10-028. PubMed: 20725109. 20725109

[7] Millar PJ, Rakobowchuk M, McCartney N, MacDonald MJ (2009) Heart rate variability and nonlinear analysis of heart rate dynamics following single and multiple Wingate bouts. Appl Physiol Nutr Metab 34: 875-883. doi:10.1139/H09-086. PubMed: 19935849. 19935849

[8] Niewiadomski W, Gasiorowska A, Krauss B, Mróz A, Cybulski G (2007) Suppression of heart rate variability after supramaximal exertion. Clin Physiol Funct Imaging 27: 309-319. doi:10.1111/j.1475-097X.2007.00753.x. PubMed: 17697028. 17697028

[9] Nakamura FY, Soares-Caldeira LF, Laursen PB, Polito MD, Leme LC et al. (2009) Cardiac autonomic responses to repeated shuttle sprints. Int J Sports Med 30: 808-813. doi:10.1055/s-0029-1234055. PubMed: 19685413. 19685413

[10] Kaikkonen P, Hynynen E, Mann T, Rusko H, Nummela A (2012) Heart rate variability is related to training load variables in interval running exercises. Eur J Appl Physiol 112: 829-838. doi:10.1007/s00421-011-2031-z. PubMed: 21678140. 21678140

[11] Franchini E, Del Vecchio FB, Matsushigue KA, Artioli GG (2011) Physiological profiles of elite judo athletes. Sports Med 41: 147-166. doi:10.2165/11538580-000000000-00000. PubMed: 21244106. 21244106

[12] Blasco Lafarga C (2009) Propuesta y resultados de una evaluación condicional específica para el entrenamiento de judo: La batería blasco aplicada en judokas españoles. Valencia: Vicerectorado de investigación y tercer ciclo; Universidad de Valencia. 296 p.

[13] Sbriccoli P, Bazzucchi I, Di Mario A, Marzattinocci G, Felici F (2007) Assessment of maximal cardiorespiratory performance and muscle power in the Italian Olympic judoka. J Strength Cond Res 21: 738-744. doi:10.1519/R-20245.1. PubMed: 17685696. 17685696

[14] Strobel G, Friedmann B, Siebold R, Bärtsch P (1999) Effect of severe exercise on plasma catecholamines in differently trained athletes. Med Sci Sports Exerc 31: 560-565. doi:10.1097/00005768-199904000-00011. PubMed: 10211852. 10211852

[15] Zouhal H, Jacob C, Rannou F, Gratas-Delamarche A, Bentué-Ferrer D et al. (2001) Effect of training status on the sympathoadrenal activity during a supramaximal exercise in human. J Sports Med Phys Fit 41: 330-336. PubMed: 11533563. 11533563

[16] Brooks GA (2007) Lactate: link between glycolytic and oxidative metabolism. Sports Med 37: 341-343. doi:10.2165/00007256-200737040-00017. PubMed: 17465603. 17465603

[17] Gladden LB (2004) Lactate metabolism: a new paradigm for the third millennium. J Physiol 558: 5-30. doi:10.1113/jphysiol.2003.058701. PubMed: 15131240. 15131240 PMC1664920

[18] Thomas, Perrey S, Lambert K, Hugon G, Mornet D et al. (2005) Monocarboxylate transporters, blood lactate removal after supramaximal exercise, and fatigue indexes in humans. J Appl Physiol 98: 804-809. PubMed: 15531559. 15531559 10.1152/japplphysiol.01057.2004 PMC2976763

[19] Thomas, Sirvent P, Perrey S, Raynaud E, Mercier J (2004) Relationships between maximal muscle oxidative capacity and blood lactate removal after supramaximal exercise and fatigue indexes in humans. J Appl Physiol 97: 2132-2138. doi:10.1152/japplphysiol.00387.2004. PubMed: 15208291. 15208291

[20] Glaister M, Stone MH, Stewart AM, Hughes M, Moir GL (2005) The influence of recovery duration on multiple sprint cycling performance. J Strength Cond Res 19: 831-837. doi:10.1519/00124278-200511000-00018. PubMed: 16331865. 16331865

[21] Glaister M (2005) Multiple sprint work : physiological responses, mechanisms of fatigue and the influence of aerobic fitness. Sports Med 35: 757-777. doi:10.2165/00007256-200535090-00003. PubMed: 16138786. 16138786

[22] Negrao CE, Middlekauff HR (2008) Adaptations in autonomic function during exercise training in heart failure. Heart Fail Rev 13: 51-60. doi:10.1007/s10741-007-9057-7. PubMed: 17932745. 17932745

[23] Rowell LB, O'Leary DS (1990) Reflex control of the circulation during exercise: chemoreflexes and mechanoreflexes. J Appl Physiol 69: 407-418. PubMed: 2228848. 2228848 10.1152/jappl.1990.69.2.407

[24] Blasco Lafarga C, Baydal Castelló E, López Rueda S, Martínez Navarro I, Carratalá Deval V et al. (2010) Validación del Test Blasco como instrumento de Evaluación Integral en judo. Ciencia, Cultura y Deporte (5): 1

[25] Nunan D, Jakovljevic DG, Donovan G, Singleton LD, Sandercock GR et al. (2010) Resting autonomic modulations and the heart rate response to exercise. Clin Auton Res 20: 213-221. doi:10.1007/s10286-010-0073-7. PubMed: 20496043. 20496043

[26] Rice SG, American Academy of Pediatrics Council on Sports M, Fitness (2008) Medical conditions affecting sports participation. Pediatrics 121: 841-848. doi:10.1542/peds.2008-0080. PubMed: 18381550. 18381550

[27] Bloomfield DM, Magnano A, Bigger JT Jr., Rivadeneira H, Parides M et al. (2001) Comparison of spontaneous vs. metronome-guided breathing on assessment of vagal modulation using RR variability. Am J Physiol Heart Circ Physiol 280: H1145-H1150. PubMed: 11179058. 11179058 10.1152/ajpheart.2001.280.3.H1145

[28] Green S, Dawson B (1993) Measurement of anaerobic capacities in humans. Definitions, limitations and unsolved problems. Sports Med 15: 312-327. doi:10.2165/00007256-199315050-00003. PubMed: 8321945. 8321945

[29] Hale T (2008) History of developments in sport and exercise physiology: AV Hill, maximal oxygen uptake, and oxygen debt. J Sports Sci 26: 365-400. doi:10.1080/02640410701701016. PubMed: 18228167. 18228167

[30] Borg G (1998) Borg's perceived exertion and pain scales. Stockholm: Human Kinetics. 104 p.

[31] Task Force of the European Society of C, the North American Society of P, Electrophysiology (1996) Heart rate variability: standards of measurement, physiological interpretation and clinical use. Circulation 93: 1043-1065

[32] Pyne DB, Boston T, Martin DT, Logan A (2000) Evaluation of the Lactate Pro blood lactate analyser. Eur J Appl Physiol 82: 112-116. doi:10.1007/s004210050659. PubMed: 10879451. 10879451

[33] Peng CK, Havlin S, Stanley HE, Goldberger AL (1995) Quantification of scaling exponents and crossover phenomena in nonstationary heartbeat time series. Chaos 5: 82-87. doi:10.1063/1.166141. PubMed: 11538314. 11538314

[34] Richman JS, Moorman JR (2000) Physiological time-series analysis using approximate entropy and sample entropy. Am J Physiol Heart Circ Physiol 278: H2039-H2049. PubMed: 10843903. 10843903 10.1152/ajpheart.2000.278.6.H2039

[35] Goldberger JJ, Le FK, Lahiri M, Kannankeril PJ, Ng J et al. (2006) Assessment of parasympathetic reactivation after exercise. Am J Physiol Heart Circ Physiol 290: H2446-H2452. doi:10.1152/ajpheart.01118.2005. PubMed: 16415073. 16415073

[36] Al Haddad H, Laursen PB, Chollet D, Ahmaidi S, Buchheit M (2011) Reliability of resting and postexercise heart rate measures. Int J Sports Med 32: 598-605. doi:10.1055/s-0031-1275356. PubMed: 21574126. 21574126

[37] Tulppo MP, Kiviniemi AM, Hautala AJ, Kallio M, Seppänen T et al. (2005) Physiological background of the loss of fractal heart rate dynamics. Circulation 112: 314-319. doi:10.1161/CIRCULATIONAHA.104.523712. PubMed: 16009791. 16009791

[38] Fukusaki C, Kawakubo K, Yamamoto Y (2000) Assessment of the primary effect of aging on heart rate variability in humans. Clin Auton Res 10: 123-130. doi:10.1007/BF02278016. PubMed: 10954070. 10954070

[39] Platisa MM, Gal V (2006) Reflection of heart rate regulation on linear and nonlinear heart rate variability measures. Physiol Meas 27: 145-154. doi:10.1088/0967-3334/27/2/005. PubMed: 16400201. 16400201

[40] Cohen J (1988) Statistical power analysis for the behavioral sciences. Hillsdale: Lawrence Erlbaum. 599 pp.

[41] Rosner B (2011) Fundamentals of Biostatistics: Brooks/Cole, Cengage. Learning.

[42] Franchini E, Takito MY, de Moraes Bertuzzi RC, Kiss M (2004) Nível competitivo, tipo de recuperação e remoção do lactato após uma luta de judô. Rev Bras Cineantrop Desempenho Hum 6: 7-16.

[43] Degoutte F, Jouanel P, Filaire E (2003) Energy demands during a judo match and recovery. Br J Sports Med 37: 245-249. doi:10.1136/bjsm.37.3.245. PubMed: 12782550. 12782550 PMC1724647

[44] Obmiński Z, Lerczak K, Witek K, Pintera M (2010) Studies on lactate peak in blood following judo match. J Combat Sports Martial Arts 2(2) Vol. 1: 95-99.

[45] Olek RA, Ziemann E, Grzywacz T, Kujach S, Luszczyk M et al. (2010) A single oral intake of arginine does not affect performance during repeated Wingate anaerobic test. J Sports Med Phys Fit 50: 52-56. 20308972

[46] Stuckey MI, Tordi N, Mourot L, Gurr LJ, Rakobowchuk M et al. (2011) Autonomic recovery following sprint interval exercise. Scand J Med Sci Sports, 22: 756–63. PubMed: 21535187. 21535187 10.1111/j.1600-0838.2011.01320.x

[47] Thomas, Cox MH, LeGal YM, Verde TJ, Smith HK (1989) Physiological profiles of the Canadian National Judo Team. Can J Sport Sci 14: 142-147. PubMed: 2819609. 2819609

[48] Leeper NJ, Dewey FE, Ashley EA, Sandri M, Tan SY et al. (2007) Prognostic value of heart rate increase at onset of exercise testing. Circulation 115: 468-474. doi:10.1161/CIRCULATIONAHA.106.666388. PubMed: 17242274. 17242274

[49] Halliwill JR, Taylor JA, Eckberg DL (1996) Impaired sympathetic vascular regulation in humans after acute dynamic exercise. J Physiol 495 ( 1): 279-288. PubMed: 8866370. 8866370 10.1113/jphysiol.1996.sp021592 PMC1160743

[50] Hynynen E, Uusitalo A, Konttinen N, Rusko H (2008) Cardiac autonomic responses to standing up and cognitive task in overtrained athletes. Int J Sports Med 29: 552-558. doi:10.1055/s-2007-989286. PubMed: 18050058. 18050058

[51] Goldstein DS, Bentho O, Park MY, Sharabi Y (2011) Low-frequency power of heart rate variability is not a measure of cardiac sympathetic tone but may be a measure of modulation of cardiac autonomic outflows by baroreflexes. Exp Physiol 96: 1255-1261. PubMed: 21890520. 21890520 10.1113/expphysiol.2010.056259 PMC3224799

[52] Reyes del Paso GA, Langewitz W, Mulder LJ, van Roon A, Duschek S (2013) The utility of low frequency heart rate variability as an index of sympathetic cardiac tone: a review with emphasis on a reanalysis of previous studies. Psychophysiology 50: 477-487. doi:10.1111/psyp.12027. PubMed: 23445494. 23445494

[53] Bosquet L, Gamelin FX, Berthoin S (2007) Is aerobic endurance a determinant of cardiac autonomic regulation? Eur J Appl Physiol 100: 363-369. doi:10.1007/s00421-007-0438-3. PubMed: 17440748. 17440748

[54] Esco MR, Olson MS, Williford HN, Blessing DL, Shannon D et al. (2010) The relationship between resting heart rate variability and heart rate recovery. Clin Auton Res 20: 33-38. doi:10.1007/s10286-009-0033-2. PubMed: 19820988. 19820988

[55] Javorka M, Zila I, Balhárek T, Javorka K (2002) Heart rate recovery after exercise: relations to heart rate variability and complexity. Braz J Med Biol Res 35: 991-1000. doi:10.1590/S0100-879X2002000800018. PubMed: 12185393. 12185393

[56] de Oliveira TP, de Alvarenga Mattos R, da Silva RB, Rezende RA, de Lima JR (2013) Absence of parasympathetic reactivation after maximal exercise. Clin Physiol Funct Imaging 33: 143-149. doi:10.1111/cpf.12009. PubMed: 23383693. 23383693

[57] Millar PJ, MacDonald MJ, McCartney N (2011) Effects of isometric handgrip protocol on blood pressure and neurocardiac modulation. Int J Sports Med 32: 174-180. doi:10.1055/s-0030-1268473. PubMed: 21165806. 21165806

[58] Ba A, Delliaux S, Bregeon F, Levy S, Jammes Y (2009) Post-exercise heart rate recovery in healthy, obeses, and COPD subjects: relationships with blood lactic acid and PaO_2_ levels. Clin Res Cardiol 98: 52-58. doi:10.1007/s00392-008-0723-0. PubMed: 18853089. 18853089

[59] Buchheit M, Duché P, Laursen PB, Ratel S (2010) Postexercise heart rate recovery in children: relationship with power output, blood pH, and lactate. Appl Physiol Nutr Metab 35: 142-150. doi:10.1139/H09-140. PubMed: 20383224. 20383224

[60] Simoes RP, Castello-Simoes V, Mendes RG, Archiza B, Santos DA et al. (2013) Lactate and Heart Rate Variability Threshold during Resistance Exercise in the Young and Elderly. Int J Sports Med. 10.1055/s-0033-1337946 23606341

[61] Tulppo MP, Kiviniemi AM, Hautala AJ, Kallio M, Seppänen T et al. (2011) Sympatho-vagal interaction in the recovery phase of exercise. Clin Physiol Funct Imaging 31: 272-281. doi:10.1111/j.1475-097X.2011.01012.x. PubMed: 21672134. 21672134

[62] Bastos FN, Vanderlei LC, Nakamura FY, Bertollo M, Godoy MF et al. (2012) Effects of cold water immersion and active recovery on post-exercise heart rate variability. Int J Sports Med 33: 873-879. doi:10.1055/s-0032-1301905. PubMed: 22722961. 22722961

[63] Buchheit M, Al Haddad H, Laursen PB, Ahmaidi S (2009) Effect of body posture on postexercise parasympathetic reactivation in men. Exp Physiol 94: 795-804. doi:10.1113/expphysiol.2009.048041. PubMed: 19395660. 19395660

[64] Miladi I, Temfemo A, Mandengué SH, Ahmaidi S (2011) Effect of recovery mode on exercise time to exhaustion, cardiorespiratory responses, and blood lactate after prior, intermittent supramaximal exercise. J Strength Cond Res 25: 205-210. doi:10.1519/JSC.0b013e3181af5152. PubMed: 20093976. 20093976

[65] Gamelin FX, Berthoin S, Bosquet L (2006) Validity of the polar S810 heart rate monitor to measure R-R intervals at rest. Med Sci Sports Exerc 38: 887-893. doi:10.1249/01.mss.0000218135.79476.9c. PubMed: 16672842. 16672842

[66] Nunan D, Donovan G, Jakovljevic DG, Hodges LD, Sandercock GR et al. (2009) Validity and reliability of short-term heart-rate variability from the Polar S810. Med Sci Sports Exerc 41: 243-250. doi:10.1249/MSS.0b013e318184a4b1. PubMed: 19092682. 19092682

[67] Wallén MB, Hasson D, Theorell T, Canlon B, Osika W (2011) Possibilities and limitations of the polar RS800 in measuring heart rate variability at rest. Eur J Appl Physiol, 112: 1153–65. PubMed: 21766225. 21766225 10.1007/s00421-011-2079-9

[68] Weippert M, Kumar M, Kreuzfeld S, Arndt D, Rieger A et al. (2010) Comparison of three mobile devices for measuring R-R intervals and heart rate variability: Polar S810i, Suunto t6 and an ambulatory ECG system. Eur J Appl Physiol 109: 779-786. doi:10.1007/s00421-010-1415-9. PubMed: 20225081. 20225081

[69] Saboul D, Pialoux V, Hautier C (2013) The impact of breathing on HRV measurements: Implications for the longitudinal follow-up of athletes. Eur J Sport Sci, 13: 1-9. PubMed: 24050471. 10.1080/17461391.2013.767947 24050471

